# Supplementary material for: Tail-tape-fused virion and non-virion RNA polymerases of a thermophilic virus with an extremely long tail
Source: Nat Commun. 2024 Jan 5;15:317. doi: 10.1038/s41467-023-44630-z (PMC10770324; doi:10.1038/s41467-023-44630-z)
Supplement: Supplementary file 1 — Supplementary information [file 41467_2023_44630_MOESM1_ESM.pdf]

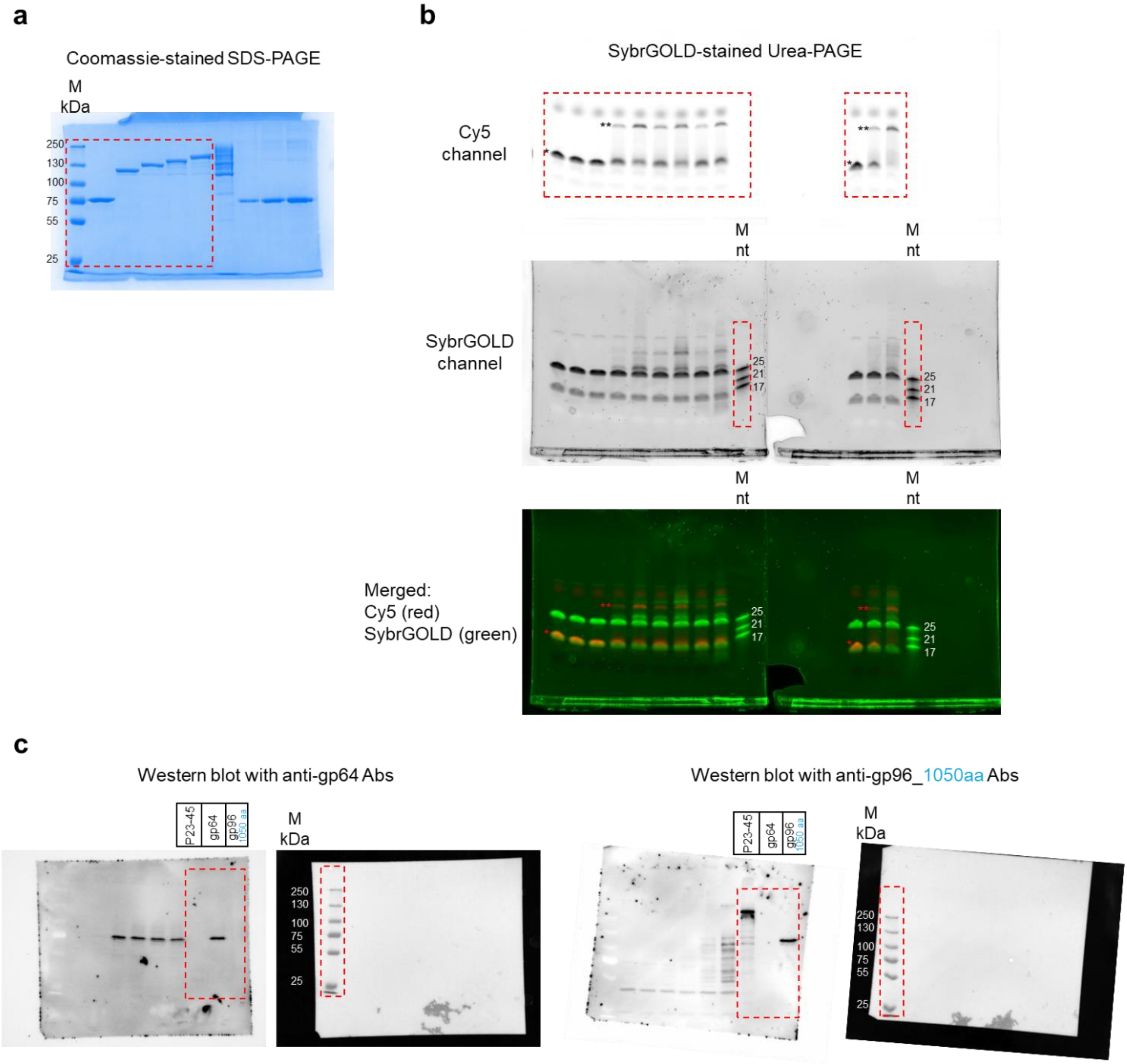

Supplementary Fig. 1: Uncropped and unedited images of gels and Western blot membranes.

SDS PAGE (a), Urea PAGE (b) and Western blot membranes (c) used in the Fig. 1 with the areas shown in the Fig. 1 marked by dashed lines. Source data are provided as a Source Data file.

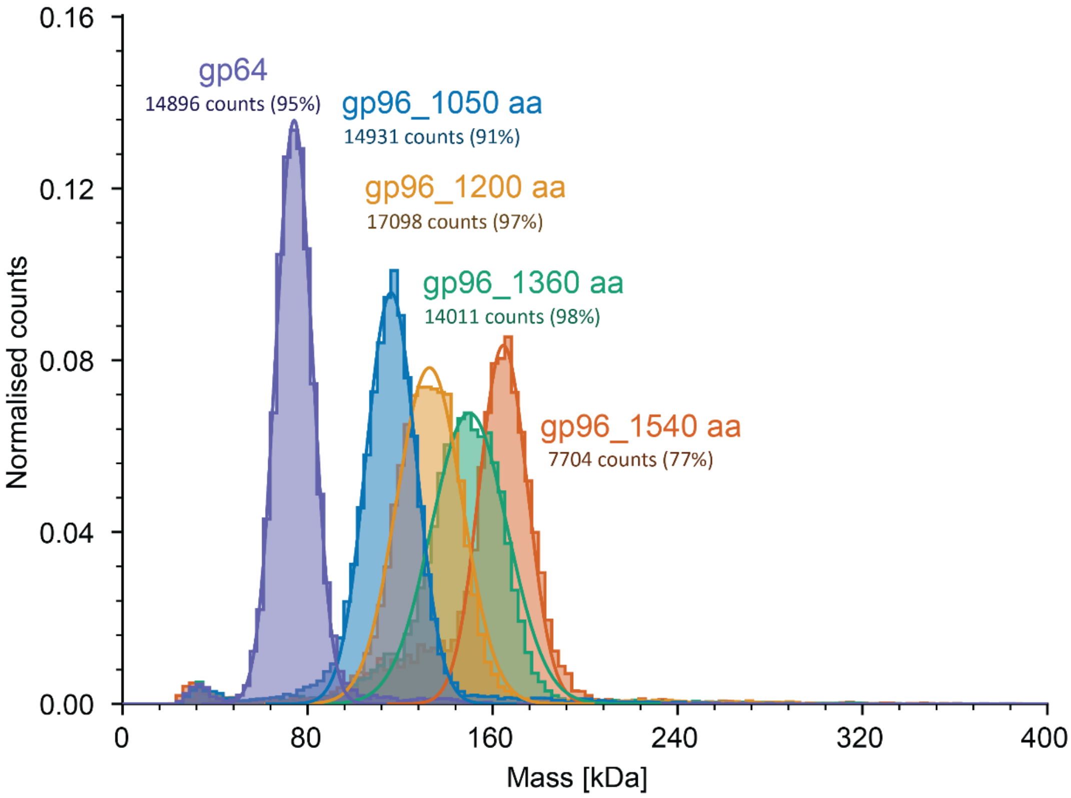

protein	expected molecular weigth	measured molecular weight ± standard deviation
gp64	68 kDa	74 kDa ± 7,9 kDa
gp96_1050 aa	118 kDa	116 kDa ± 10,8 kDa
gp96_1200 aa	134 kDa	133 kDa ± 14,1 kDa
gp96_1360 aa	152 kDa	150 kDa ± 16,5 kDa
gp96_1540 aa	172 kDa	165 kDa ± 10,4 kDa

Supplementary Fig. 2: Mass photometry analysis of virion and non-virion P23-45 RNAPs. Molecular mass distributions of the virion RNAP (fragments of gp96) and non-virion RNAP (gp64) determined by mass photometry are shown on top. Each protein was measured in triplicates. Expected and measured molecular weights are listed in the table at the bottom. All proteins are present in solution as monomers. Source data are provided as a Source Data Mass Photometry file.

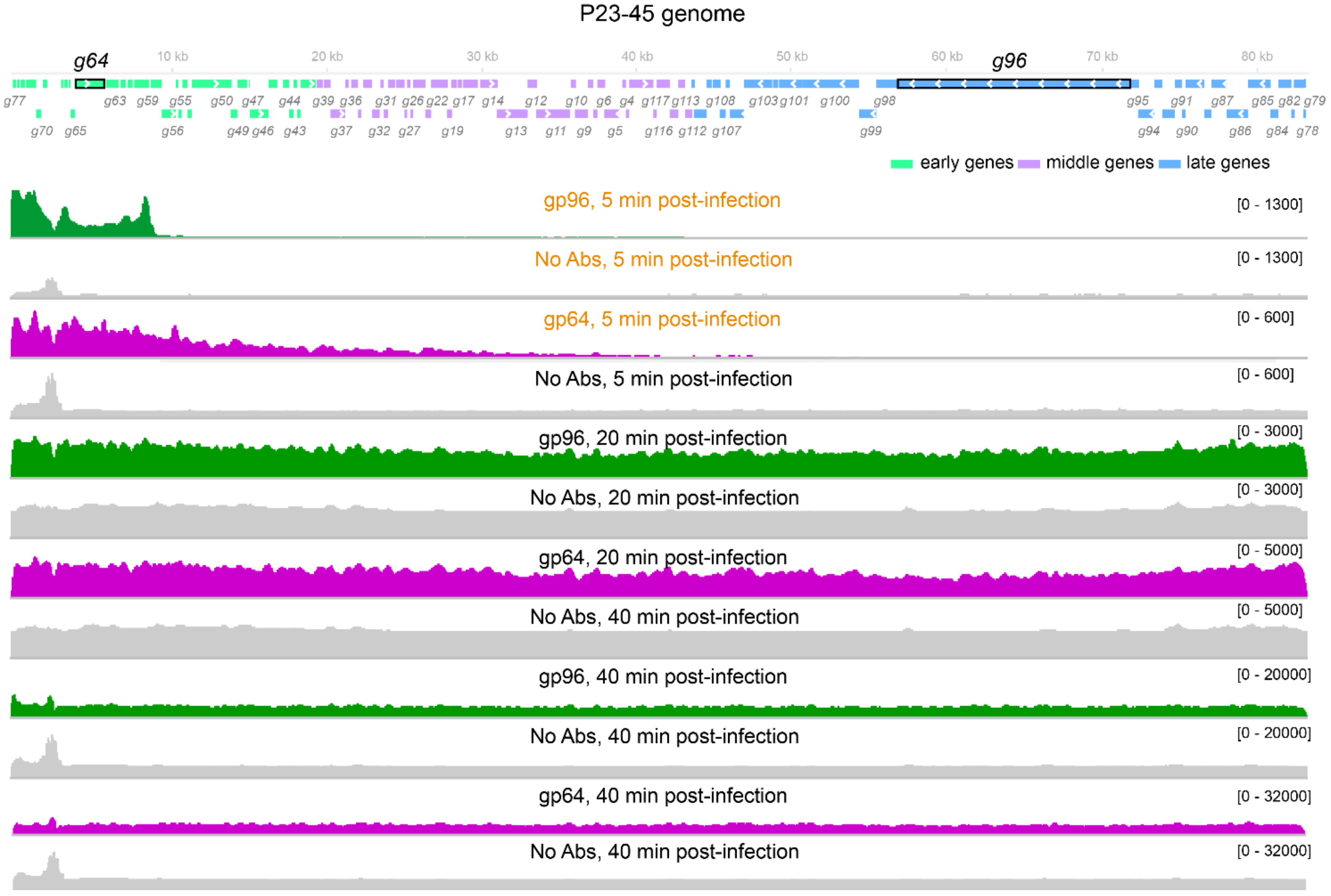

Supplementary Fig. 3: Distribution of gp96 and gp64 RNAPs along P23-45 genome. Coverage tracks of DNA co-immunoprecipitated from *T. thermophilus* culture 5, 20, and 40 minutes post-infection with P23-45 are shown. Antibodies raised against the 1540-residue fragment of gp96 and against gp64 were used in the experiment. Tracks shown in Fig. 2 are indicated by orange-colored text.

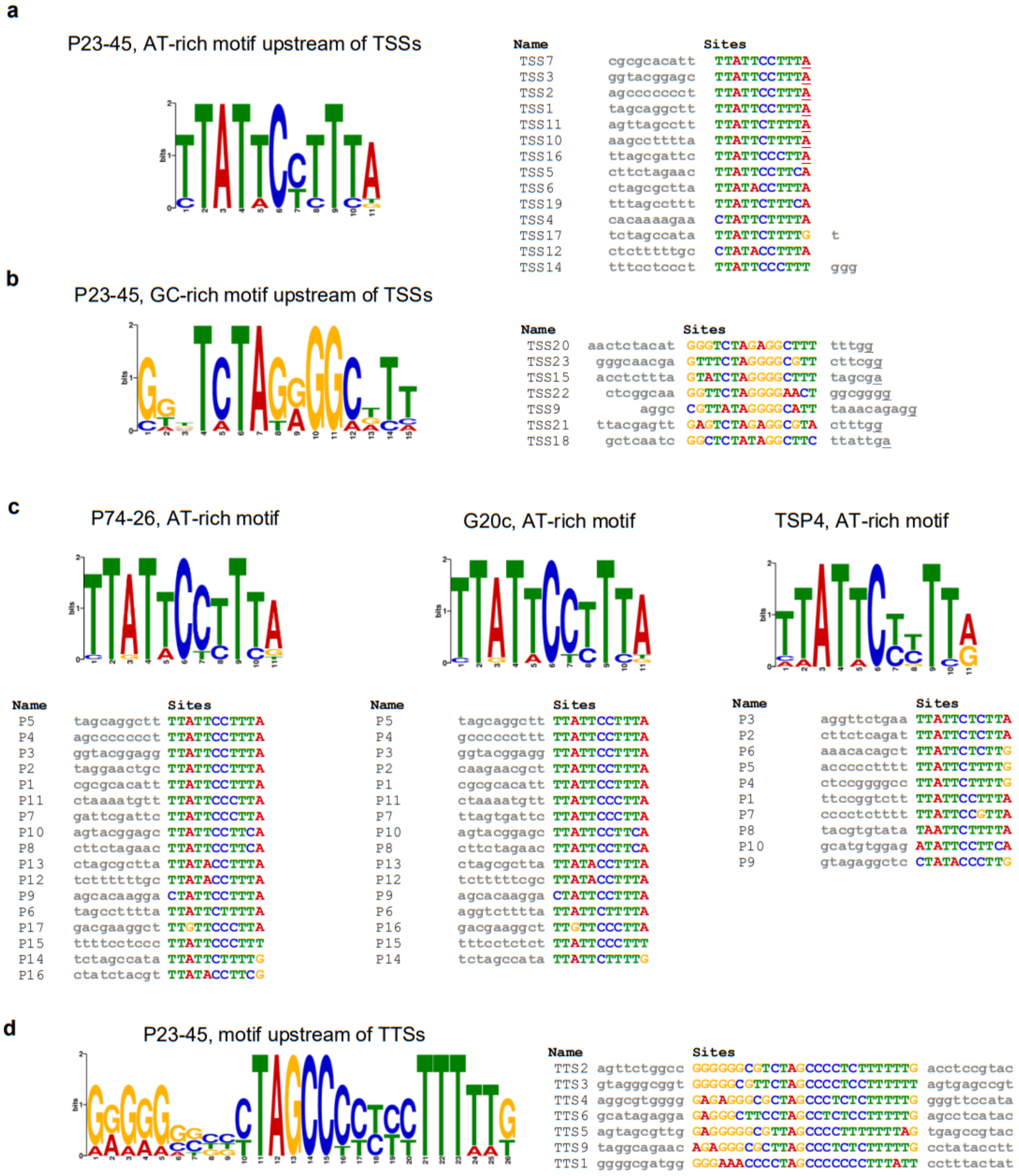

Supplementary Fig. 4: Conserved motifs in the genomes of P23-45 and related phages. a, b, Sequence logos of AT-rich (a) and GC-rich (b) motifs revealed by MEME[1] upstream of P23-45 transcription start sites (TSSs) identified through the ONT-cappable-seq (Supplementary Data 1,2). c, sequence logos of AT-rich motifs discovered by FIMO[2] in the genomes of phages P74-26, G20c, and TSP4 using the P23-45 AT-rich motif as an input. d, Sequence logo of a

motif discovered by MEME[1] in front of transcription termination sites (TTSs) identified through the ONT-cappable-seq (Supplementary Data 1). Source data are provided as a Source Data file.

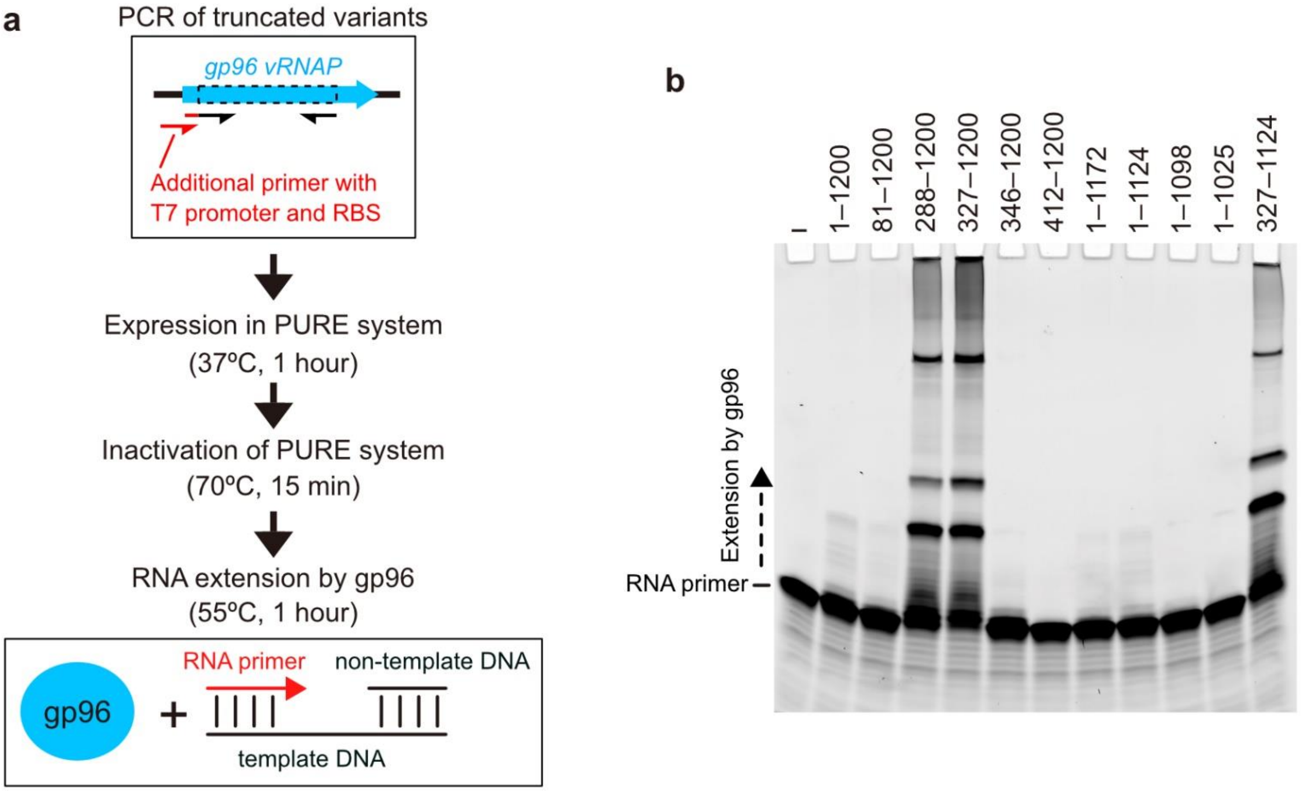

Supplementary Fig. 5: Cell-free activity analysis of P23-45 gp96 vRNAP. a, Schematic depiction of the cell-free activity test of truncated gp96 variants. b, RNA primer elongation by truncated gp96 variants. The 327–1,124 fragment is the shortest active variant identified in this experiment, which mostly corresponds to the ordered regions in the crystal of the 1–1,200 fragments (Supplementary Fig. 6). Source data are provided as a Source Data file.

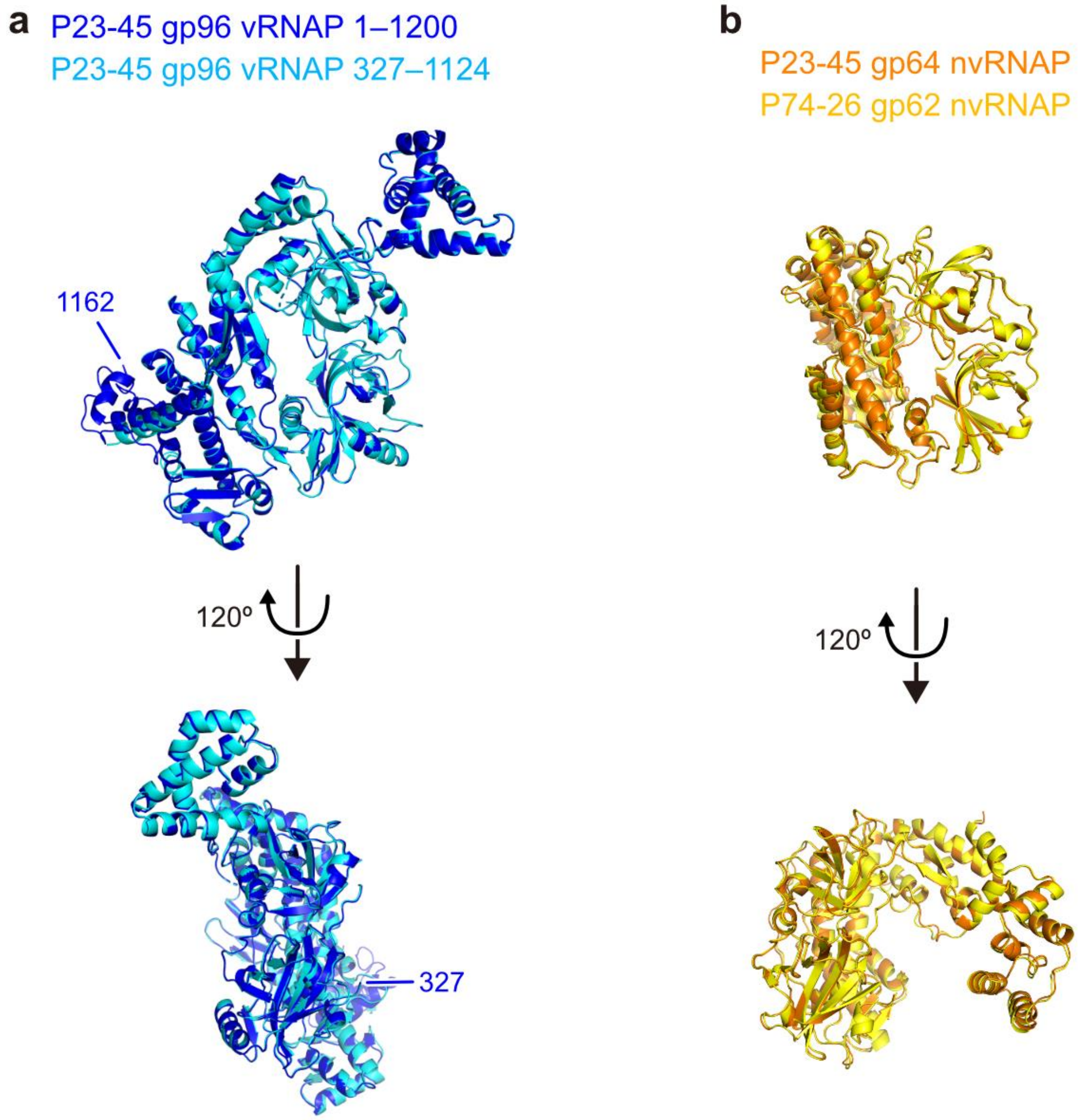

Supplementary Fig. 6: Crystal structures of *Thermus thermophilus* phage RNAPs. a, The crystal structures of the 1–1,200 and 327–1,124 fragments of P23-45 gp96 vRNAP are superimposed (blue: 1–1,200; cyan: 327–1,124). b, The crystal structures of P23-45 gp64 nvRNAP and P74-26 gp62 nvRNAP are superimposed (orange: P23-45 gp64; yellow: P74-26 gp62).

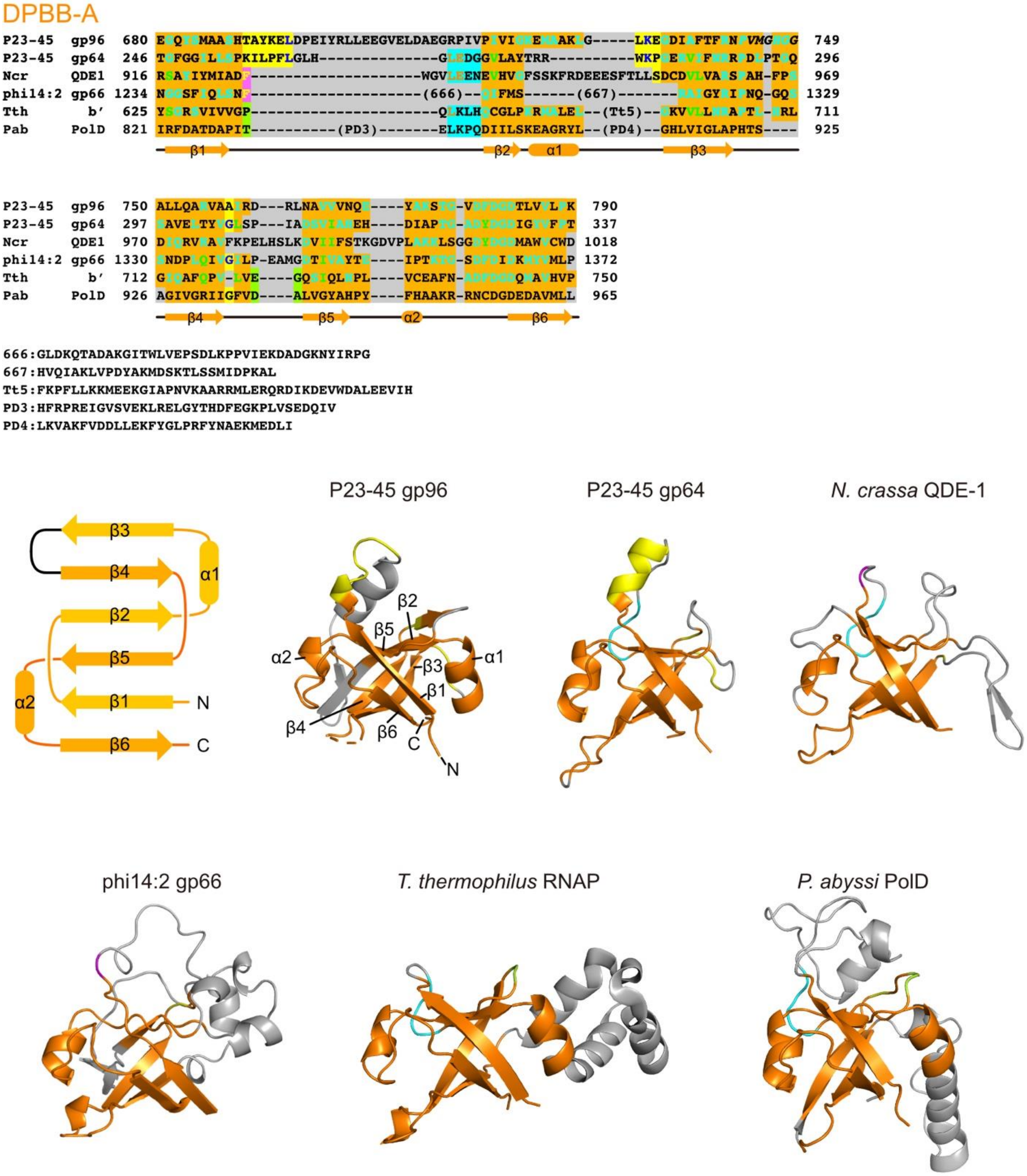

Supplementary Fig. 7: Structures of the DPBB-A domains from different two-barrel RNAPs. The structure-based alignment (top) and local structures (middle and bottom) of the DPBB-A domains of P23-45 gp96 vRNAP, P23-45 gp64 nvRNAP, *N. crassa* QDE-1 (PDB: 2J7N[3]), phi14:2 gp66 (PDB: 6VR4[4]), *T. thermophilus* RNAP (PDB: 2O5J[5]), and *P. abyssi* PolD (6T8H[6]) are shown. The topology diagram of the DPBB fold is also shown (middle left). The same color scheme as in Fig. 3 is applied. Local structures specifically conserved between *T. thermophilus* RNAP and *P. abyssi* PolD are additionally colored limon. The disordered region

of P23-45 gp96 vRNAP (743–749) is shown in italic font in the structural alignment and as a dashed line in the ribbon model. The DPBB domains have a topology with pseudo-twofold symmetry, comprised of two α-helices and six β-strands.

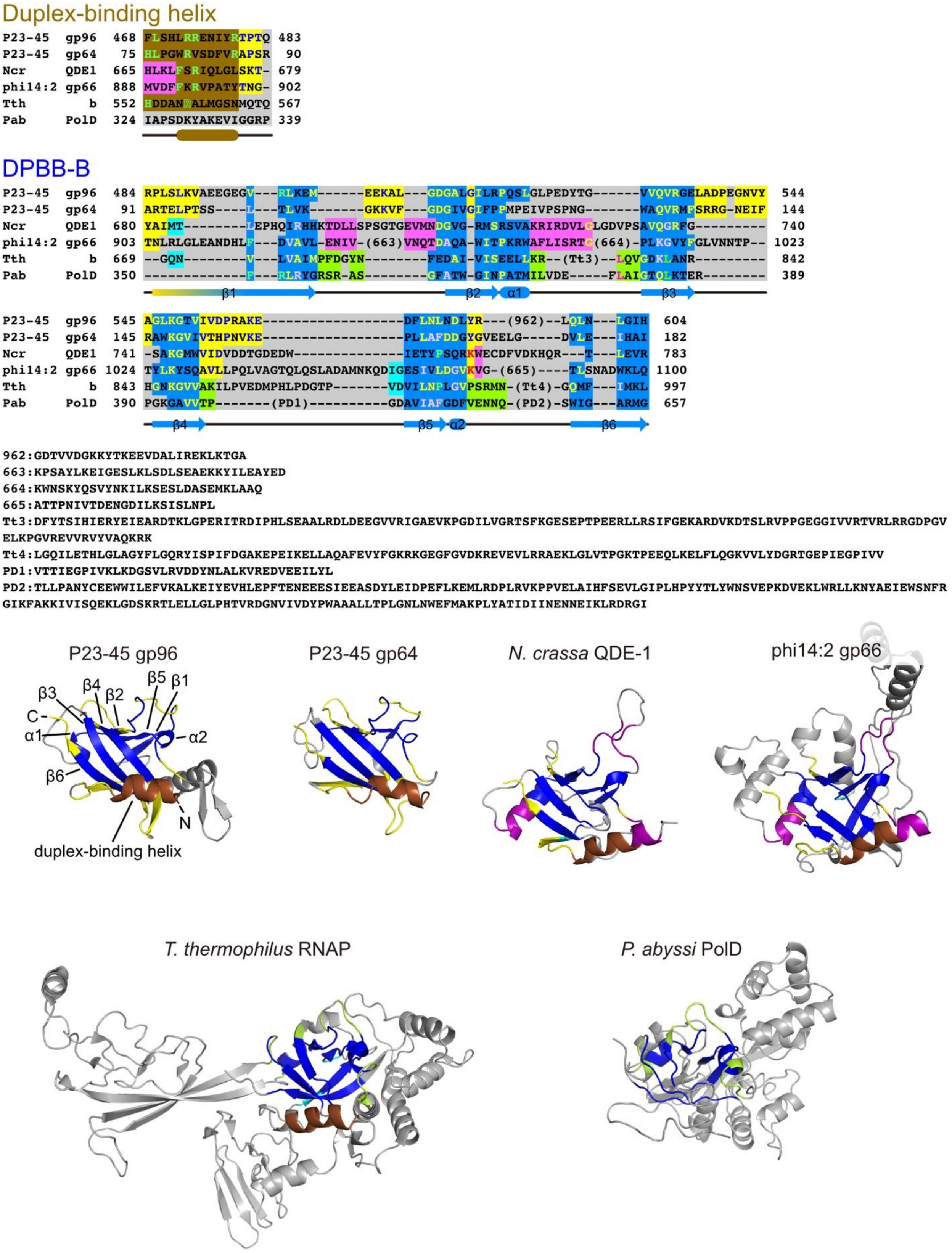

Supplementary Fig. 8: Structures of the DPBB-B domains from different two-barrel RNAPs. The structure-based alignment (top) and local structures (bottom) of the duplex-binding helices and DPBB-B domains of P23-45 gp96 vRNAP, P23-45 gp64 nvRNAP, *N.*

crassa QDE-1 (PDB: 2J7N[3]), phi14:2 gp66 (PDB: 6VR4[4]), *T. thermophilus* RNAP (PDB: 2O5J[5]), and *P. abyssi* PolD (6T8H[6]) are shown. Long lineage-specific insertions are shown below the alignment. The same color scheme as in Fig. 3 is applied. Local structures specifically conserved between *T. thermophilus* RNAP and *P. abyssi* PolD are additionally colored limon. The loops connecting β1/β2, β3/β4, and β4/β5 are conserved in the two P23-45 RNAP (yellow). The α1 helix is extended in QDE-1 and phi14:2 gp66 (purple). The loop connecting β1/β2 is partially conserved in QDE-1 and phi14:2 gp66, although phi14:2 gp66 has a longer insertion.

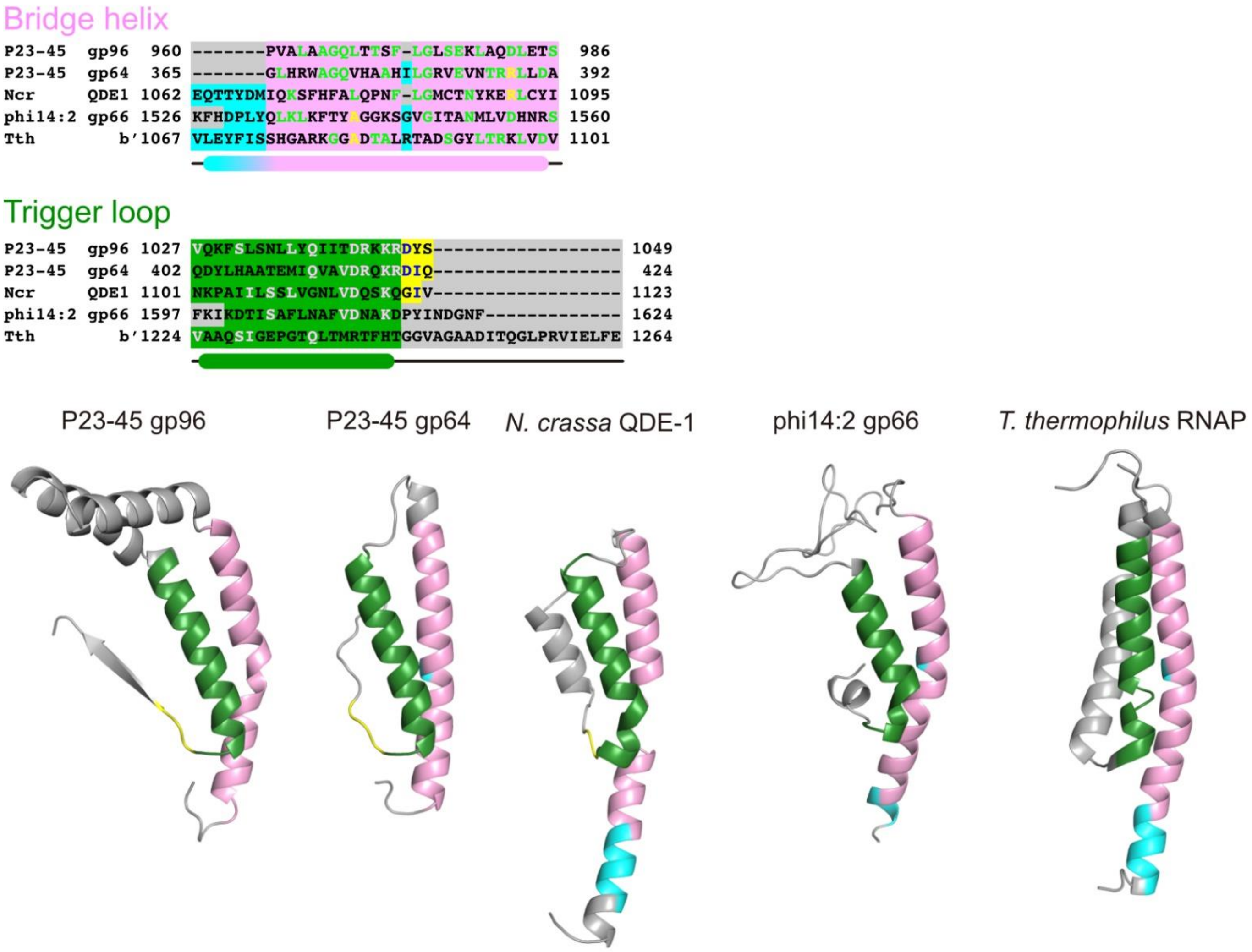

Supplementary Fig. 9: Structures of the bridge helices and trigger loops of different two-barrel RNAPs. The structure-based alignment (top) and local structures (bottom) of the bridge helices and trigger loops of P23-45 gp96 vRNAP, P23-45 gp64 nvRNAP, *N. crassa* QDE-1 (PDB: 2J7N[3]), phi14:2 gp66 (PDB: 6VR4[4]), and *T. thermophilus* RNAP (PDB: 2O5J[5]) are shown. The same color scheme as in Fig. 3 is applied. The structure of the bridge helix (pink) is conserved except for extensions at the N-terminus (QDE-1, phi14:2 gp66, and *T. thermophilus* RNAP) or C-terminus (gp64). The N-terminal part of the trigger loop (green) is also conserved. The C-terminal part of the trigger loop is not conserved.

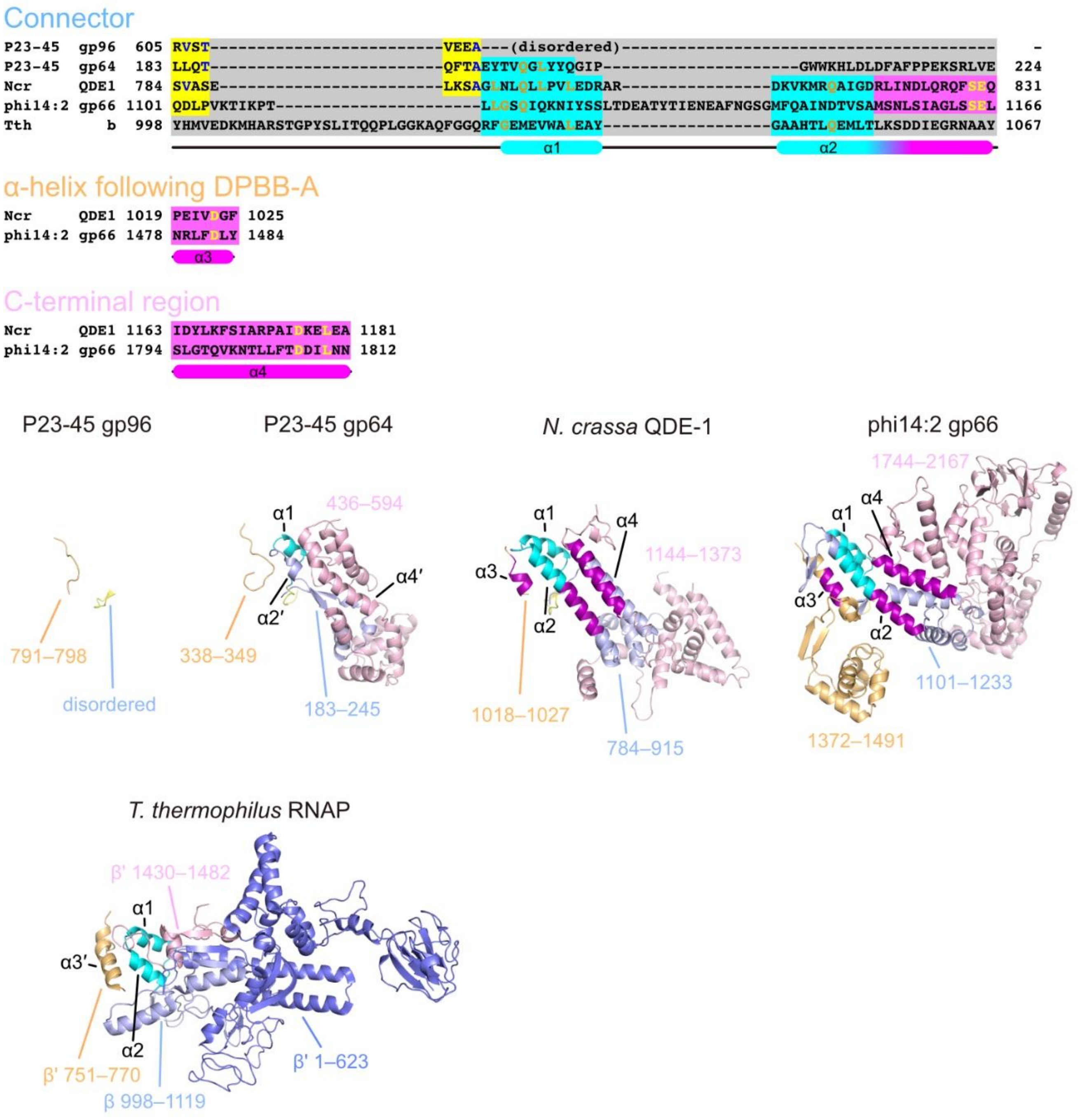

Supplementary Fig. 10: Structures of the clamp domains of different two-barrel RNAPs. The structure-based alignment (top) and local structures (bottom) of the clamp domains of P23-45 gp96 vRNAP, P23-45 gp64 nvRNAP, *N. crassa* QDE-1 (PDB: 2J7N[3]), phi14:2 gp66 (PDB: 6VR4[4]), and *T. thermophilus* RNAP (PDB: 2O5J[5]) are shown. The structure-based alignments for the α-helix following DPBB-A and the C-terminal regions contain only limited parts of QDE-1 and phi14:2 gp66, as other RNAPs or structural elements could not be aligned confidently. The clamp domains are comprised of three parts (the connector between DPBB-B and DPBB-A – light blue; the α-helix following DPBB-A – light orange; the C-terminal region

– light pink). The conserved regions are colored according to the color code in Fig. 3. The connector part of *T. thermophilus* RNAP contains the C-terminal region of the β subunit (light blue) and the N-terminal part of the β′ subunit (slate), which are fused in some bacteria.

The connector regions of gp64 nvRNAP, QDE-1, phi14:2 gp66, and *T. thermophilus* RNAP share an α-helical part (cyan: α1, 2), although α2 is not conserved in gp64 nvRNAP (α2′). The α2 helix is further extended in QDE-1 and phi14:2 gp66 (purple). Although the α-helix following DPBB-A and the C-terminal regions have diverged, QDE-1 and phi14:2 share a few helices (α3, 4). *T. thermophilus* RNAP has an α-helix in a similar position to α3 (α3′), but their precise alignment was not possible by manual structure comparison. Gp64 also has an α-helix in a similar position to α4 (α4′). However, their evolutionary relationship is unclear as the structures surrounding the α-helix α4′ in gp64 are largely different from QDE-1 and phi14:2 gp66. In the gp96 vRNAP structure, the short connector region is disordered, and the C-terminal region and the α-helix following DPBB-A are absent.

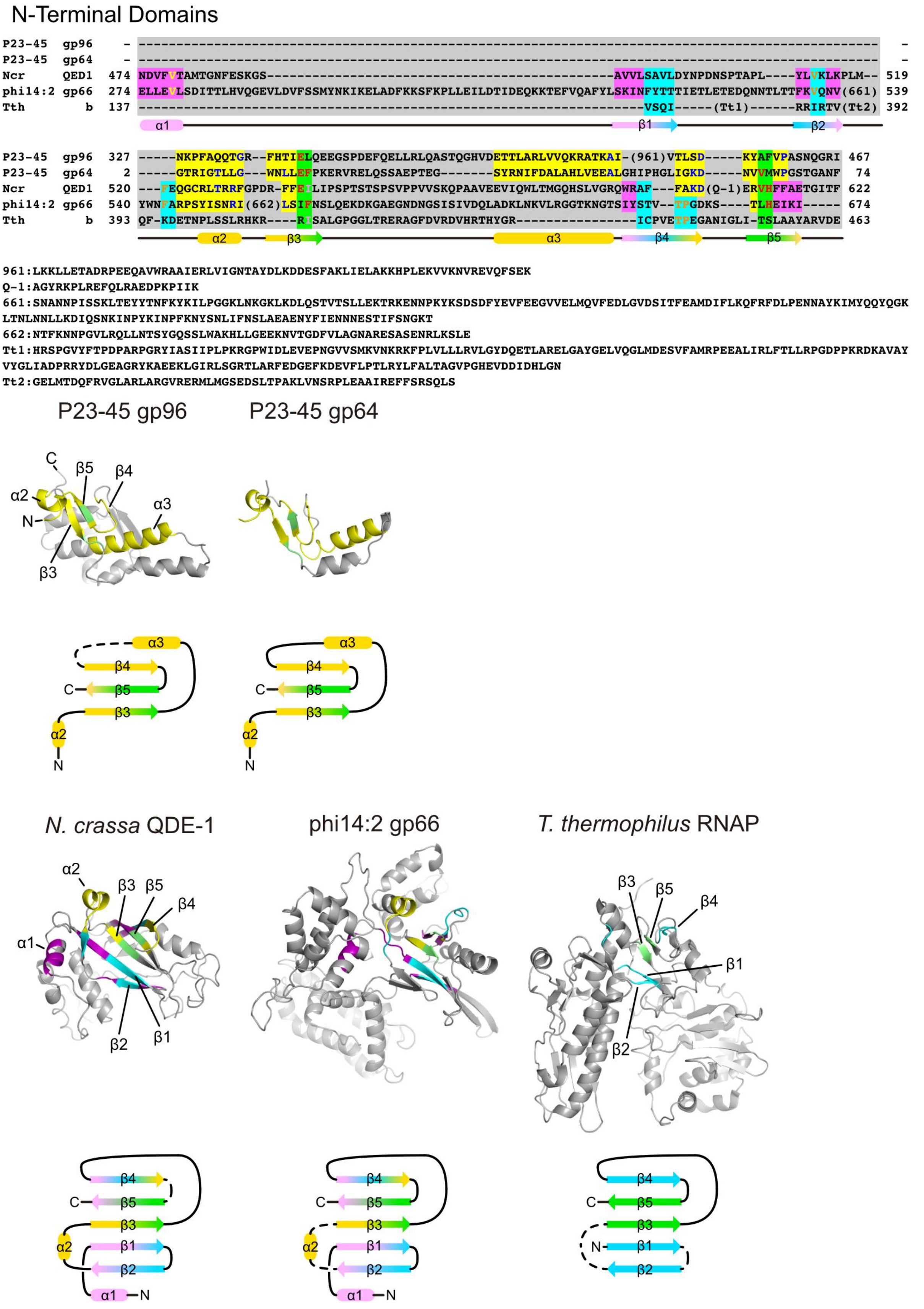

Supplementary Fig. 11: Structures of the N-terminal domains from different two-barrel RNAPs. The structure-based alignment (top), local structures (middle), and the topology

diagrams (bottom) of the N-terminal domains of P23-45 gp96 vRNAP, P23-45 gp64 nvRNAP, *N. crassa* QDE-1 (PDB: 2J7N[3]), phi14:2 gp66 (PDB: 6VR4[4]), and *T. thermophilus* RNAP (PDB: 2O5J[5]) are shown. Long lineage-specific insertions are shown below the alignment. The same color scheme as in Fig. 3 is applied. The N-terminal regions of the single subunit RNAPs partially share a common structure and have likely diverged from a common ancestor. The N-terminal domains of the two P23-45 RNAPs contain a common motif comprised of two α-helices (α2, 3) and three β-strands (β3–5), although gp96 vRNAP has a more extended structure. The motif can also be found in QDE-1 and phi14:2 gp66 RNAP in a modified form with one α-helix lost (α3) and another α-helix (α1) and two β-strands (β1, 2) added, indicating the N-terminal domains of single-subunit RNAPs emerged from a common ancestor and diverged into two distinct classes. Although the N-terminal structure of the β subunit of *T. thermophilus* RNAP does not align well with others, the strand order and direction of its β sheet are the same as the corresponding parts of QDE-1 and gp66, which might indicate their common origin.

Supplementary Table 1. X-ray data collection and atomic structure refinement statistic for the gp96 crystals.

	1–1200 (PDB: 8H2M)	327–1124 (PDB: 8H2N)
**Data collection**		
Space group	$P4_122$	$P2_1$
Cell dimensions		
a, *b*, *c* (Å)	137.8, 137.8, 135.4	117.9, 128.5, 136.4
α, β, γ (°)	90, 90, 90	90, 96.9, 90
Wavelength	0.978 Å	0.979 Å
Resolution (Å)	50–3.08 (3.27–3.08)[1] *	50–4.41 (4.52–4.41)
R_{merge} (%)	92.5 (-)	24.3 (198.6)
R_{meas} (%)	93.0 (-)	28.4 (231.8)
$I / \sigma I$	14.7 (0.0)	4.08 (0.67)
CC ½	0.998 (0.475)	0.989 (0.191)
Completeness (%)	100 (100)	99.5 (98.4)
Redundancy	86.9 (76.5)	3.5 (3.6)
Unique reflections	46152 (7612)[2]	50313 (3691)[2]
**Refinement**		
Resolution (Å)	50–3.08	50–4.41
No. reflections	46100[2]	50147[2]
R_{work} / R_{free}	0.205/0.254	0.245/0.303
No. atoms	5938	22384
Protein	5938	22380
Ligand/ion	0	4
Water	0	0
B-factors	79.0	203.3
Protein	79.0	203.3
Ligand/ion	-	148.3
Water	-	-
R.m.s. deviations		
Bond lengths (Å)	0.012	0.003
Bond angles (°)	1.465	0.671

*Values in parentheses are for highest-resolution shell.

[1]Datasets from eight crystals were automatically processed and merged by KAMO. The high-resolution cutoff was also determined automatically.

[2]Anomalous diffractions.

Supplementary Table 2. **X-ray data collection and atomic structure refinement statistic for the gp62 and gp64 crystals**

	Native gp62 (PDB: 8F5M)	SeMet gp62	Native gp64 (PDB: 8F72)
**Data collection**			
Space group	$C222_1$	$P2_12_12_1$	$C2_1$
Cell dimensions			
a, *b*, *c* (Å)	110.9, 210.1, 181.1	115.6, 122.3, 122.7	210.3, 106.3, 112.4
α, β, γ (°)	90, 90, 90	90, 90, 90	90, 90, 90
Wavelength (Å)	0.97856	0.97856	0.97856
Resolution (Å)*	50.0-2.38 (2.42-2.38)	50.0-2.8 (2.9-2.8)	50.0-2.7 (2.8-2.7)
R_{sym} or R_{merge}	10.7 (129.5)	7.7 (66.1)	7.0 (73.1)
$I / \sigma I$	13.7 (1.9)	26.1 (1.6)	12.3 (2.15)
Completeness (%)	100 (99.9)	98.2 (84.4)	99.7 (99.9)
Redundancy	8.0 (8.1)	6.5 (4.4)	4.2 (4.2)
**Refinement**			
Resolution (Å)	25.0-2.4		25.0-2.7
No. reflections	74,361		63,058
R_{work} / R_{free}	19.4/23.7		20.4/25.5
No. atoms			
Protein	9,317		9,335
Ligand/ion	2		2
Water	831		146
B-factors			
Protein	40.1		82.0
Ligand/ion	45.6		67.8
Water	43.2		38.1
R.m.s. deviations			
Bond lengths (Å)	0.008		0.005
Bond angles (°)	1.3		1.34

*One crystal was used for each data set reported.
*Values in parentheses are for highest-resolution shell.

Supplementary Table 3. The primers and PCR schemes for the cell-free analysis.

gp96 fragment	Forward primer 1 (× 1)	Forward primer 2 (× 0.1)	Reverse primer (× 1)
1–1200	T7RNAP-RBS: GCGAATTAATACGACTCACTATAGGGCTTAAGTATAAGGAGGAAAAAATATGAAAATAAAAACAGGAGCACGCAATAAC	1200F2-1: ATGAAAATAAAAACAGGAGCACGCAATAACatggacctggagagata	1200R3-1200: ggcctgtacagaattcggtccTTATTAcctagccacctcgtccc
81–1200	T7RNAP-RBS	1200F2-81: ATGAAAATAAAAACAGGAGCACGCAATAACgaagtgcgcgagctggcca	1200R3-1200
288–1200	T7RNAP-RBS	1200F2-288: ATGAAAATAAAAACAGGAGCACGCAATAACaaaggcgcaagttgggaa	1200R3-1200
327–1200	T7RNAP-RBS	1200F2-327: ATGAAAATAAAAACAGGAGCACGCAATAACaacaaacctttcgcccagcaa	1200R3-1200
346–1200	T7RNAP-RBS	1200F2-346: ATGAAAATAAAAACAGGAGCACGCAATAACggctctcccgacgagtttca	1200R3-1200
412–1200	T7RNAP-RBS	1200F2-412: ATGAAAATAAAAACAGGAGCACGCAA	1200R3-1200

		TAACacggcgtacgatttga agga	
1–1172	T7RNAP-RBS	1200F2-1	1200R3-1172: ggcctgtacagaattcggtccT TATTAagaaccttgcggctt gttcggc
1–1124	T7RNAP-RBS	1200F2-1	1200R3-1124: ggcctgtacagaattcggtccT TATTAatccgggtcctcctt
1–1098	T7RNAP-RBS	1200F2-1	1200R3-1098: ggcctgtacagaattcggtccT TATTAcggaaccctttcctct acgg
1–1025	T7RNAP-RBS	1200F2-1	1200R3-1025: ggcctgtacagaattcggtccT [illegible]
327–1124	T7RNAP-RBS	1200F2-327	1200R3-1124

1. Bailey TL, Elkan C. Fitting a mixture model by expectation maximization to discover motifs in biopolymers. *Proc Int Conf Intell Syst Mol Biol* **2**, 28-36 (1994).

2. Grant CE, Bailey TL, Noble WS. FIMO: scanning for occurrences of a given motif. *Bioinformatics* **27**, 1017-1018 (2011).

3. Salgado PS, Koivunen MR, Makeyev EV, Bamford DH, Stuart DI, Grimes JM. The structure of an RNAi polymerase links RNA silencing and transcription. *PLoS Biol* **4**, e434 (2006).

4. Drobysheva AV*, et al.* Structure and function of virion RNA polymerase of a crAss-like phage. *Nature* **589**, 306-309 (2021).

5. Vassylyev DG, Vassylyeva MN, Zhang J, Palangat M, Artsimovitch I, Landick R. Structural basis for substrate loading in bacterial RNA polymerase. *Nature* **448**, 163-168 (2007).

6. Madru C*, et al.* Structural basis for the increased processivity of D-family DNA polymerases in complex with PCNA. *Nat Commun* **11**, 1591 (2020).
