# Supplementary material for: Tail-tape-fused virion and non-virion RNA polymerases of a thermophilic virus with an extremely long tail
Source: Nat Commun. 2024 Jan 5;15:317. doi: 10.1038/s41467-023-44630-z (PMC10770324; doi:10.1038/s41467-023-44630-z)
Supplement: Supplementary file 3 — Description of Additional Supplementary Files [file 41467_2023_44630_MOESM3_ESM.docx]

**Description of Additional Supplementary Files**

**Supplementary Data 1:**

Transcription start and termination sites in the P23-45 genome identified through ONT-cappable-seq

**Supplementary Data 2:**

Conserved AT-rich motif identified in P23-45 related phages

**Supplementary Data 3:**

Alignments of distinct RNAP families used to build UPGMA dendrogram

**Supplementary Data 4:**

Viruses with two or more RNAP polypeptide per genomes (identified by PSI-BLAST search and filtered by presence of "[RK]xPx{20,100}DxDxD" motif)

**Supplementary Data 5:**

Number of reads in ChIP-seq experiment

**Supplementary Data 6:**

Sequencing quality, read lengths, and mapping metrics for ONT-cappable-seq
